# Longitudinal proteome-wide antibody profiling in Marburg virus survivors identifies wing domain immunogen for vaccine design

**DOI:** 10.1038/s41467-024-51021-5

**Published:** 2024-09-17

**Authors:** Surender Khurana, Gabrielle Grubbs, Supriya Ravichandran, Emily Cluff, JungHyun Kim, Ana I. Kuehne, Samantha Zak, John M. Dye, Julius J. Lutwama, Andrew S. Herbert

**Affiliations:** 1 Division of Viral Products, Center for Biologics Evaluation and Research (CBER), FDA, Silver Spring, MD 20993 USA; 2 Virology Division, U.S. Army Medical Research Institute of Infectious Diseases, Fort Detrick, Frederick, MD USA; 3 Department of Arbovirology, Emerging, and Re-emerging Infection, Uganda Virus Research Institute, Entebbe, Uganda

**Keywords:** Viral infection, Marburg virus, Antibodies

## Abstract

Limited knowledge exists on the quality of polyclonal antibody responses generated following Marburg virus (MARV) infection and its evolution in survivors. In this study, we evaluate MARV proteome-wide antibody repertoire longitudinally in convalescent phase approximately every six months for five years following MARV infection in ten human survivors. Differential kinetics were observed for IgM vs IgG vs IgA epitope diversity, antibody binding, antibody affinity maturation and Fc-receptor interaction to MARV proteins. Durability of MARV-neutralizing antibodies is low in survivors. MARV infection induces a diverse epitope repertoire with predominance against GP, VP40, VP30 and VP24 that persisted up to 5 years post-exposure. However, the IgM and IgA repertoire declines over time. Within MARV-GP, IgG recognize antigenic sites predominantly in the amino-terminus, wing domain and GP2-heptad repeat. Interestingly, MARV infection generates robust durable FcɣRI, FcɣRIIA and FcɣRIIIA IgG-Fc receptor interactions. Immunization with immunodominant MARV epitopes reveals conserved wing region between GP1 and GP2, induces neutralizing antibodies against MARV. These findings demonstrate that MARV infection generates a diverse, long-lasting, non-neutralizing, IgG antibody repertoire that perturbs disease by FcɣR activity. This information, along with discovery of neutralizing immunogen in wing domain, could aid in development of effective therapeutics and vaccines against Marburg virus.

## One sentence summary

Longitudinal Marburg virus human antibody repertoire

## Abstract (web summary)

There is limited understanding of long-term antibody dynamics after Marburg virus infection. Here, Khurana et al report the antibody responses in Marburg virus disease survivors over five years and identify a conserved immunogen within the virus wing domain that could serve as a target for vaccine design.

## Introduction

Marburg virus (MARV) is a member of Filoviridae family that causes severe and often fatal disease in humans. There is currently no licensed vaccine or therapeutic treatment against Marburg virus disease (MVD). The recent outbreak of highly pathogenic MARV in Equatorial Guinea has 15 confirmed MVD cases and 23 probable cases (https://www.who.int/emergencies/disease-outbreak-news/item/2023-DON459). The areas reporting cases are about 150 kilometers apart, suggesting wider transmission of the virus, and most likely an under-reported number of total cases. The >80% high case-fatality ratio with 11 deaths among the laboratory confirmed, and demise of all probable cases, have highlighted the paucity of available effective medical countermeasures against MVD^1^. The recent spread to Tanzania in March 2023, with 8 confirmed MVD cases and 5 deaths, suggests the possibility of more-widespread circulation, and has raised concerns of a global public health emergency. This follows several large outbreaks in 1998-2000 and 2004-2005 in the Democratic Republic of the Congo (DRC) and Angola, respectively, and multiple outbreaks in Uganda from 2007-2017, with a > 80% mortality rate. Bats serve as a natural host and viral reservoir of MARV and the virus is mostly transmitted via direct or indirect contact with blood, secretions, organs, or bodily fluids or via surfaces contaminated with these fluids through broken skin or mucous membranes^2,3^. Filoviruses infects and affects multiple organ systems and tissues in exposed humans. The virus can persist in immune-privileged sites like semen, uterus, eye, placenta, breast milk etc. for years in some survivors^3–7^. Consequently, it is feared that latent MARV can be transmitted via sexual transmission and transplacental transmission, with the result that MARV outbreaks can be re-ignited, resulting in severe epidemics. Therefore, the development of effective vaccines and therapeutics against MARV is considered a global public health priority^1^.

Since passive transfer of antibodies in animal models is known to protect against lethal MARV challenge, it is postulated that humoral immune response generated following MARV infection in survivors may provide protection against MVD^8–10^. Given recurrent MVD outbreaks in Africa and an ongoing risk for regional and international spread, it remains unclear if prior MARV infection will provide long-term immunity against MVD. Primarily, ELISA and MARV-neutralization tests have been used to analyze the anti-MARV antibodies^11^, but these assays provide limited insight into the diversity and quality of polyclonal antibody responses across the complete MARV proteome and its evolution over time in survivors.

In this study, we performed a comprehensive analysis of the quality and durability of the humoral immune response following MARV infection in MVD survivors. Longitudinal in-depth quantitative and qualitative immune-profiling of polyclonal plasma were performed to elucidate binding and neutralizing antibodies, and antibody epitope repertoires using GFPDL and SPR technology to measure real-time antibody binding kinetics, antibody affinity, and IgG-Fc receptor activity across the complete MARV proteome during convalescence from 12-months for up to 5 years following MARV infection in 10 MVD survivors and 10 matched uninfected local controls from the 2012 MARV outbreak in Uganda^12^. To identify potential vaccine and therapeutic targets against MARV, we investigated the potential of immunodominant antigenic sites identified across the MARV proteome using GFPDL to generate MARV binding and neutralizing antibodies.

## Results

### Durable virion binding but non-neutralizing antibodies against Marburg virus in longitudinal samples from MVD survivors

**Fig. 1:**
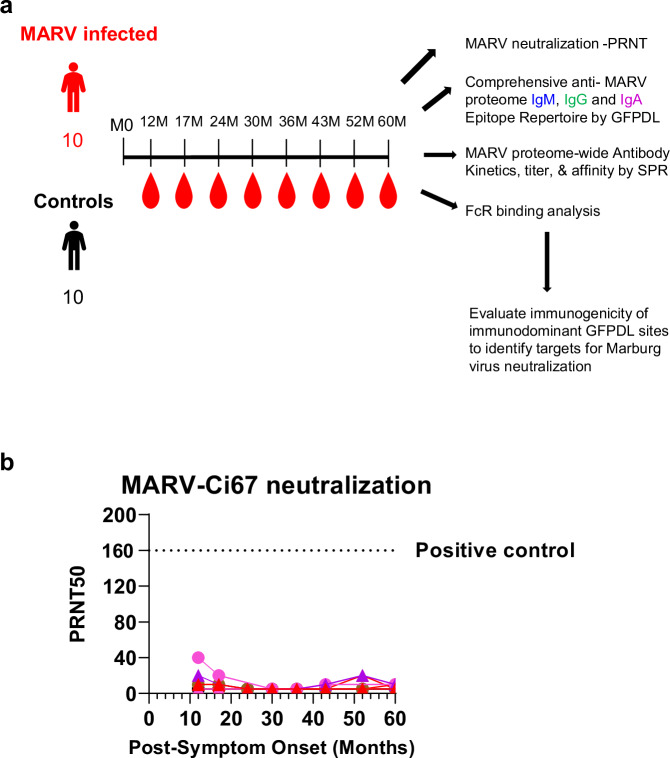
MARV binding and neutralizing antibodies in MVD survivors. **a** Overview of cohort with longitudinal convalescent samples collected approximately every six months from 10 MVD survivors and 10 matched uninfected local cohort from the 2012 Uganda outbreak. Comprehensive antibody profiling of each plasma sample was performed via Marburg virion neutralizing antibodies in PRNT, MARV-proteome wide IgM, IgG, IgA epitope repertoire by GFPDL, recombinant MARV protein binding antibody kinetics using surface plasmon resonance and Fc-receptor interactions in Luminex assay. **b** Neutralizing titer (PRNT50) against wild-type MARV Ci67 are shown for the 10 MVD survivors (in colors) and 10 uninfected controls (in black did not show any detectable virus neutralization). A positive control human serum sample demonstrated neutralization activity against MARV with a PRNT50 titer of 1:160 and is depicted as dashed line. Source data are provided as a Source Data file.

Comprehensive antibody repertoire profiling was performed on individual plasma samples collected approximately every six months for up to 5 years from 10 survivors of the 2012 Uganda MARV outbreak^11^ (Table 1) during the convalescent phase (at least ∼9 months after the disease resolution), with all timepoints being relative to the months post-infection/exposure (Fig. 1a). Health questionnaires completed by participating survivors indicated no reports of any sequelae that could be attributed to MARV infection nor any subsequent diagnosis of ‘recurrent MARV’ in the years following initial infection. All participants reported being healthy and free of illness at all collection timepoints. None of the survivors received antibody or small molecule therapies following exposure. In addition to survivor samples, plasma was collected from 10 uninfected local matched controls.

**Table 1:** Study cohort characteristics

Patient ID	Gender	Age range at time of infection (years)	Hospitalization
MK1	Female	21–30	2 weeks
MK2	Male	21–30	4 weeks
MK3	Female	11–20	3 weeks
MK4	Male	21–30	2 weeks
MK5	Male	31–40	4 days
MK6	Female	41–50	2 weeks
MK7	Female	Unknown	Unknown
MK8	Female	21–30	2 weeks
MK9	Female	21–30	4 days
MK10	Female	Unknown	1.5 weeks

The samples were tested for neutralizing antibodies against a wild-type MARV-Ci67 strain by plaque reduction neutralization test (PRNT) in a BSL-4 laboratory. Very low neutralizing antibodies (50% neutralization titer; PRNT50 ≤ 1:20) against MARV-Ci67 strain were observed in all survivors at all evaluated timepoints during convalescence which is near the limit of detection for the PRNT (Fig. 1b). The positive control human serum sample demonstrated a PRNT50 titer of 1:160 against MARV. As expected, all 10 uninfected controls did not contain any neutralizing antibodies against MARV.

### MARV infection generates a diverse proteome-wide IgM, IgG, and IgA epitope repertoire in survivors

To determine the evolution of polyclonal IgM, IgG, and IgA epitope repertoire following MARV infection in survivors across the entire MARV proteome, MARV-GFPDL containing sequences ranging from 50-1000 bp long from the complete MARV genome with >10^8.1^ unique phage clones was subjected to affinity selection with longitudinal samples collected at 12-, 30-, 43- and 60-months post-symptom onset. The MARV-GFPDL displayed linear and conformational (secondary and possibly tertiary but not quaternary) epitopes with random distribution of size and sequence of inserts that spanned the complete MARV proteome (Supplementary Fig. S1). The MARV-GFPDL adsorbed 91% of MARV-GP specific IgG in the MVD survivor’s polyclonal human sample supporting the use of the MARV-GFPDL for antibody epitope repertoire analysis of post-infection human samples (Supplementary Fig. S2).

Due to limitations of the sample volumes available in this longitudinal plasma collection, the primary goal was to identify the overall pattern of antibody epitope diversity in pooled plasma samples at various timepoints following human MARV infection in MVD survivors as a whole rather than to determine fine antibody specificities in each individual plasma sample. Previous studies with SARS-CoV-2 and EBOV in humans demonstrated that the antibody epitope profile in pooled samples was overall similar to the epitope fingerprint determined for individual samples^13,14^. The pooled sample from 10 uninfected (MARV-negative) control adults bound <20 phages from the MARV-GFPDL. For the pooled post-MARV infection sample from 10 MVD survivors, the number of bound phages ( ~ 2 x 10e4) for IgM and IgG were 3 -fold higher than IgA (8 x 10e3) at the 12-month timepoint (Supplementary Fig. S3). The number of IgG MARV-specific phage titers remained consistent over time for up to 5 years (Supplementary Fig. S3). However, the number of GFPDL phages recognized by MARV-specific IgM and IgA declined by 48-fold and 273-fold from 12-months to 60 months post-exposure in these MVD survivors, respectively (Supplementary Fig. S3).

**Fig. 2:**
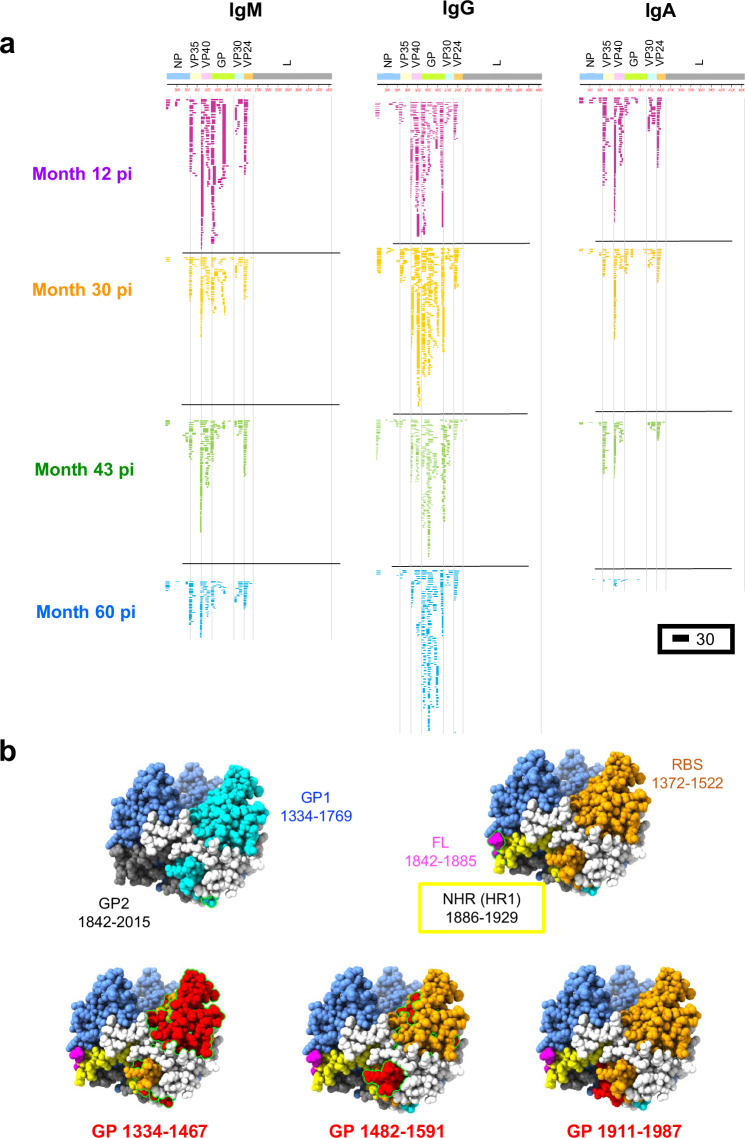
Evolution of MARV proteome-wide IgM, IgG, and IgA antibody epitope repertoires in MVD survivors. **a** IgM, IgG, and IgA antibody epitope repertoire recognized in the human plasma at different months post-MARV infection (pi) during convalescence (12-, 30-, 43- and 60-months pi are color coded) and their alignment to the whole proteome of MARV (showing different proteins: NP, VP35, VP40, GP, VP30, VP24 and L). Graphical distribution of representative clones with a frequency of ≥2, obtained after affinity selection, are shown. The horizontal position and the length of the bars indicate the peptide sequence displayed on the selected phage clone to its homologous sequence in the MARV proteome on alignment. The thickness of each bar represents the frequency of repetitively isolated phage, with the scale shown below the alignment. The GFPDL affinity selection data was performed in duplicate (two independent experiments by researcher in the lab, who was blinded to sample identity), and similar number of phage clones and epitope repertoire was observed in both phage display analysis. **b** Structural representations of immunodominant antigenic sites recognized by IgG using MARV-GFPDL on the surface structure of mature trimeric MARV GP solved structure [PDB #5UQY^44^]. The different domains of mature GP: GP1 (residues 1334-1769 in cyan), receptor binding site (RBS; residues 1372-1522 in orange), GP2 (residues 1842-2015 in blue), fusion loop (FL; residues 1842-1885 in pink) and N-terminal heptad repeat (NHR; residues 1886-1929 in yellow) are shown on the structure of MARV-GP, with the residue numbers corresponding to the complete MARV proteome used for GFPDL. The MARV GP structure used for crystallography encodes for MARV GPΔmuc ectodomain (encompassing amino acid residues 1–636 with a mucin deletion of residues 257–425). The immunodominant antigenic sites recognized by IgG in MVD survivors identified using MARV-GFPDL that can be mapped on the crystal structure are depicted in red.

MARV sequences expressed by phages bound by post-infection IgM showed a diverse epitope repertoire distribution, displaying small and large sequences spanning the entire MARV proteome, apart from the L polymerase (Fig. 2a and Supplementary Fig. S4 that shows the data without the L protein). At 12-months post-infection, IgM antibodies recognized antigenic sites in N-terminal of VP35, VP30 and VP24, multiple sites within matrix protein (VP40) and GP, and a few sites within nucleoprotein (NP). At 30-months and 43-months post-infection, IgM epitope profile remained diverse with marginal decline in antibody binding to sites in NP, VP35, VP40, and GP. By 60-months post-infection, although the total number of IgM bound phages declined, IgM repertoire remained diverse across the MARV proteome, with IgM recognizing multiple epitopes in VP35, VP40, and N-terminus of GP in these survivors (Fig. 2a).

The diverse IgG epitope repertoire at 12-months post-exposure in MVD survivors recognized epitopes at the N-terminus of NP, VP35, and VP24, multiple immunodominant sites in VP40 and GP, and a few epitopes in VP30 (Fig. 2a). At five years post-MARV infection, the phage titers bound to IgG, as well as the diversity of epitopes recognized across the MARV proteome, were durable, with the persistence of epitopes in the N-terminus of NP, VP35, and VP24, and immunodominant sites in VP40 and GP.

MARV GP is an important target for development of vaccines and therapeutic antibodies. Within GP, the IgG predominantly recognized epitopes in the N-terminus of the receptor binding site (RBS) and in the C-terminus of GP1 at 12 months (Supplementary Fig. S5), which were also recognized by the IgM in these MVD survivors. In addition, the IgG in 12-months post-infection samples recognized an immunodominant site at the C-terminus of GP2 encompassing the C-terminal heptad repeat (CHR; residues 1950-1967 in complete MARV proteome) and the membrane proximal external region (MPER; residues 1968-1989 in complete MARV proteome). Over time, the antibody diversity within GP expanded in these MVD survivors to other sites including the C-terminal half of GP1, as well as in the N-terminus of GP2. The highest frequency of IgG molecules targeted the C-terminus of GP2 epitope encompassing the CHR and the MPER, which were long-lasting and observed from 12 to 60 months post-infection in these survivors. The GFPDL identified immunodominant antigenic sites recognized by the IgG in MVD survivors were exposed on the surface of the MARV GP structure (Fig. 2b). The predominantly recognized IgG antigenic sites in other MARV structural proteins were also surface exposed and are shown on the structures of NP, VP35, VP40, VP30 and VP24 (Supplementary Fig. S6).

MARV infection generated an equally diverse IgA antibody repertoire that recognized antigenic sites across the MARV proteome defined by multiple epitopes in VP40 and VP30 as well as at the N-terminus of VP35, VP24, and GP at 12-months post-exposure in these survivors (Fig. 2a). However, unlike IgG, the IgA phage titers and epitope diversity declined over time such that by 5 years post-exposure, very few IgA antibodies recognized MARV epitopes (Fig. 2a and Supplementary Fig. S3).

### Differential evolution of antibody profile against MARV proteins in MVD survivors

To elucidate the quantitative and qualitative antibody reactivity of each individual plasma from survivors to full length proteins coded by the MARV genome, we analyzed serial dilutions of individual polyclonal plasma samples against purified recombinant proteins corresponding to the MARV proteome using Surface Plasmon Resonance (representative sensorgram for plasma serial dilutions against MARV-GP is shown in Supplementary Fig. S7). Antibody binding measured in SPR is independent of secondary antibody and determines total combined antibody binding due to all isotypes (IgM+IgG+IgA) in the polyclonal plasma to the MARV protein captured on the sensor chip.

**Fig. 3:**
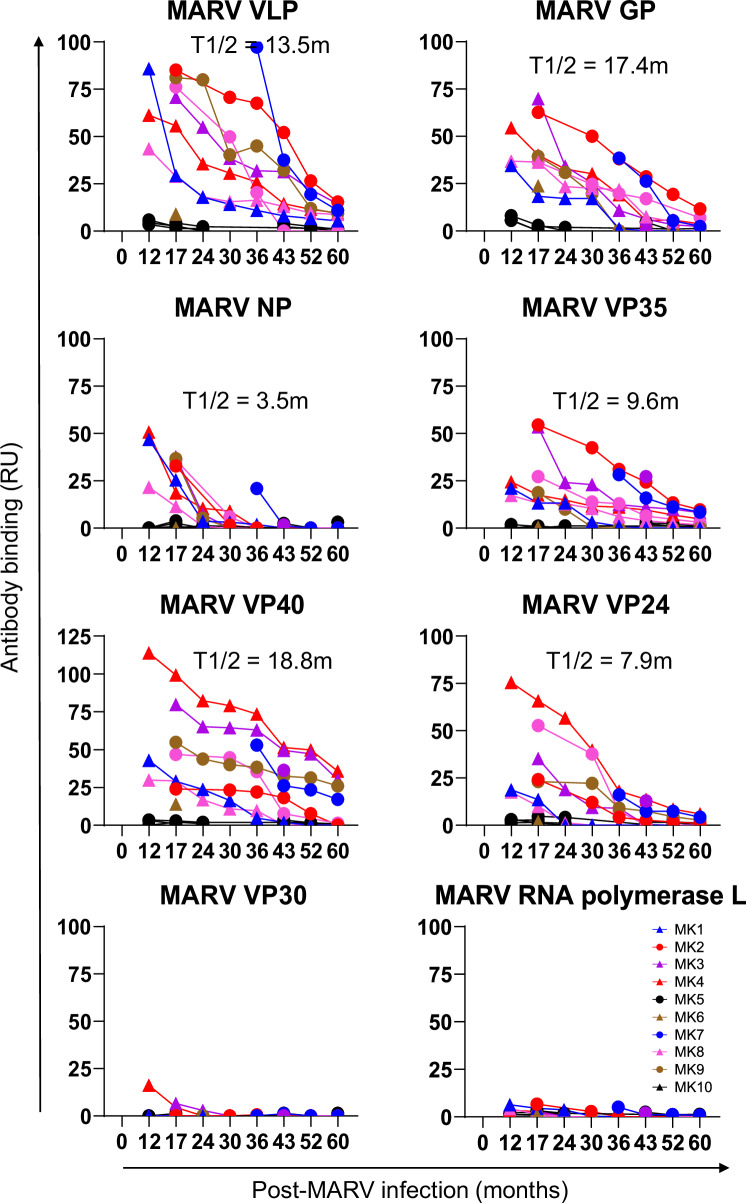
Longitudinal SPR based analysis of MVD survivor plasma against purified recombinant MARV proteins. Serial dilutions of longitudinal plasma samples collected at 12-months through 60-months post-infection from the MVD survivors (*N* = 10; in colors) and uninfected controls (*N* = 10; in black) were analyzed for antibody binding to purified recombinant MARV proteins by SPR. Total antibody binding is represented in SPR resonance units (RU) for 10-fold dilution of plasma sample binding to Marburg virus-like particles (VLPs expressing trimeric GP on the surface of particles made of MARV matrix proteins VP40 and NP), and recombinant MARV GP, NP, VP35, VP40, VP24, VP30 and L polymerase. The mean half-life (in months) of the polyclonal binding antibodies against various MARV proteins for the convalescent phase in these MVD survivors is shown, except for VP30 and L, which demonstrated weak antibody binding. All SPR experiments were performed twice and the researchers performing the assay were blinded to sample identity. The variation for each sample in duplicate SPR runs was <7%. The data shown is the average value of two experimental runs. Source data are provided as a Source Data file.

Minimal or absence of antibody binding ( < 10 resonance unit) was observed for 10 uninfected control plasma against various MARV proteins in SPR (Fig. 3). At the 12-month timepoint for each of the MVD survivors, the highest antibody binding titers were observed to the Marburg virus-like particles (VLPs) displaying trimeric GP on the surface of the VLP (VLP expressing trimeric GP on the surface of particles made of MARV matrix proteins VP40 and NP), and to purified recombinant MARV VP40, followed by GP, VP35, VP24, and NP, with minimal antibody binding to VP30 and no binding to L polymerase (Fig. 3). The antibody binding to various MARV proteins declined in the MVD survivors over time such that by 60-months post-infection there was moderate to weak antibody binding to most MARV proteins by SPR. It is worth noting that antibody decay over time observed by SPR is a measure of total antibody binding to MARV proteins and is a combination of all isotypes (IgM+IgG+IgA) and may mimic the combinatorial effect of the IgG+IgM+IgA phage titers observed by the MARV-GFPDL approach (Supplementary Fig. S3). We calculated the half-life (T1/2) for antibody decay against various MARV proteins for the convalescent phase in these MVD survivors. The mean half-life of polyclonal binding antibodies against various MARV proteins ranged from 3.5 months against NP, to 18.8 months against VP40, for the convalescent phase in these MVD survivors (Fig. 3).

To measure antibody affinity maturation following MARV infection, the antibody-antigen complex dissociation rates (off-rate constants) were determined as a surrogate for the average affinity of polyclonal antibody binding using SPR^13–15^. Technically, since plasma antibodies are bivalent or multivalent, the proper term for their binding is avidity, but here we use the term affinity throughout since we measured primarily monovalent interactions, as was shown in previous studies^13,16,17^. Antibody off-rate constants, which describe the fraction of antigen-antibody complexes that decay per second and are independent of antibody concentration, were determined directly from the plasma antibody interaction with MARV proteins in the dissociation phase only for the sensorgrams with maximum RU in range of 10-100 RU in the optimized SPR (Supplementary Fig. S7). Antibody affinity was not determined for 10 uninfected control samples against any MARV protein or for the MVD survivors against MARV-VP30 or L, as most of the post-infection plasma showed very low antibody binding ( < 10 RU) against these two proteins.

**Fig. 4:**
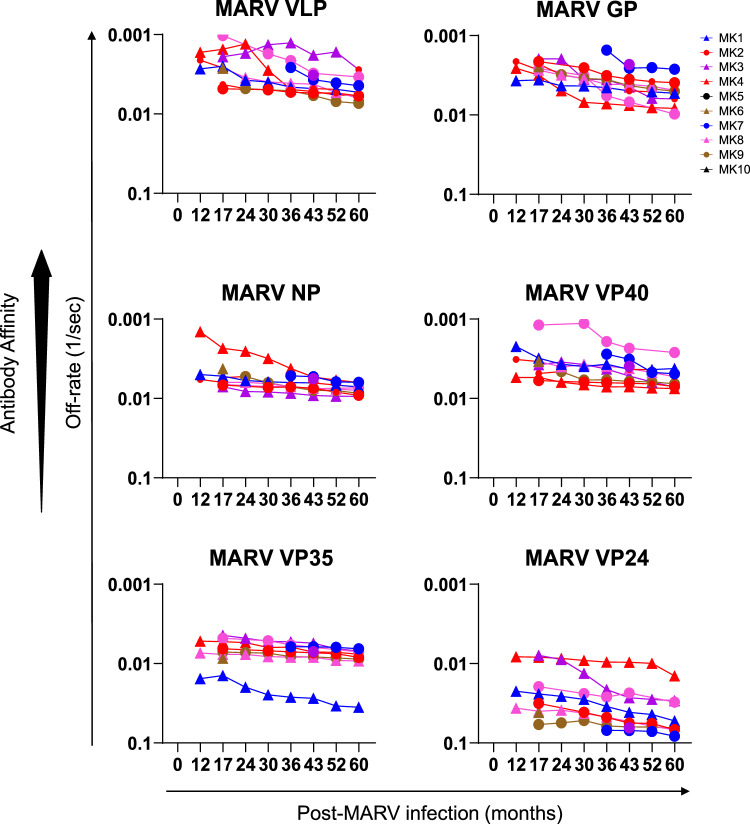
Antibody affinity maturation of MVD survivor plasma against MARV proteome. Antibody affinity maturation of longitudinal plasma samples collected from MVD survivors to purified recombinant MARV proteins was determined by SPR. Antibody off-rate constants that describe the fraction of antibody-antigen complexes decaying per second were determined directly from the interaction of serially diluted post-infection plasma with MARV proteins using SPR in the dissociation phase only for the sensorgrams with maximum RU in the range of 10-100 RU in the optimized SPR. Antibody affinity was not determined for uninfected control samples or against MARV-VP30 or L as most of the post-infection MVD plasma samples showed very low antibody binding ( < 10 RU) against these two proteins. All SPR experiments were performed twice and the researchers performing the assay were blinded to sample identity. The variation for each sample in duplicate SPR runs was <7%. The data shown is the average value of two experimental runs. Source data are provided as a Source Data file.

The off-rates of polyclonal plasma antibodies bound to most of the MARV proteins ranged between 0.01-0.001/second, indicating moderate affinity generated following MARV infection in these MVD survivors, while the antibodies against VP24 (off rate >0.01/sec) were of lower affinity (Fig. 4). At 12 months post-infection, median off-rate of plasma antibodies to MARV VLP (0.0021/sec), GP (0.00258/sec) and VP40 (0.00322/sec) were slower (indicating stronger antibody affinity) followed by antibody affinity against MARV NP (0.00499/sec) and MARV VP35 (0.00733/sec), and weakest affinity (faster off-rate) against VP24 (0.0223/sec) in the 10 MVD survivors. By five years post-infection, the antibody affinity declined (faster off-rate) but only marginally (1 to 3-fold reduction) against these MARV proteins (median off-rate: VLP; 0.00558/sec, GP; 0.00518/sec, VP40; 0.00592/sec, NP; 0.00762/sec, VP35; 0.00798/sec and VP24; 0.0585/sec). Taken together, these observations suggest that MARV infection generates persistent, long-lasting, moderate affinity antibodies that are primarily of IgG isotype in MVD survivors.

### Long-lasting IgG-Fc-receptor interactions following MARV infection in MVD survivors

In these MVD survivors, we observed anti-MARV binding IgG that did not neutralize wild-type MARV Ci67 strain in the classical PRNT assay. Neutralization by antibodies is often the main mechanism to prevent or reduce viral infection. However, there are other antibody-effector functions mediated by Fc and Fc-domain sensors on adaptive and innate immune cells such as antibody-dependent cellular cytotoxicity (ADCC) and antibody-dependent cellular phagocytosis (ADCP), that act either independently or in combination with neutralization to reduce viral disease or infection^18–20^. ADCC and ADCP could contribute to viral particles clearance through interaction with FcγRIIIA and FcγRI/FcγRIIA, respectively^21–23^. FcγRI, FcγRIIA, and FcγRIIIA are all activating receptors, with FcγRI being the only FcγR with high-affinity, while the rest of FcγR, having medium to low affinity to IgG-Fc^24^. As each FcγR is associated with a specific effector function, determining the FcγR interaction with MARV-specific antibody binding can be a good surrogate marker to evaluate the potential of antibody Fc-mediated protection against MVD.

**Fig. 5:**
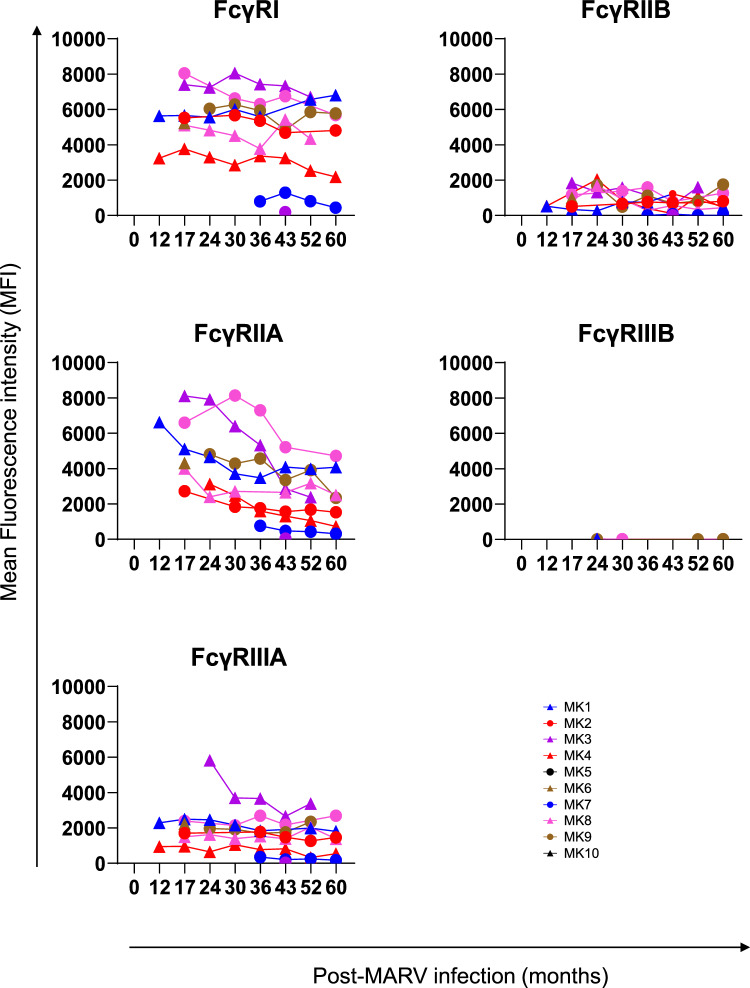
Longitudinal assessment of FcγRs interaction with post-MARV infection plasma. Quantification of FcγRI, FcγRIIA, FcγRIIIA, FcγRIIB, or FcγRIIIB interaction with longitudinal 10 MVD survivor plasma antibodies from 12 to 60 months post-infection bound to MARV VLP (recombinant VLPs expressing MARV trimeric GP on the surface of particles made of MARV matrix proteins VP40 and NP) coupled beads using the bead-based assay. The level of binding is shown as mean fluorescent intensity (MFI). Each sample was run in duplicate, and each dot represents an average of duplicate values. The variation for each sample in duplicate runs was <5%. Source data are provided as a Source Data file.

To understand if anti-MARV IgG in the plasma of these survivors has the capacity to mediate various Fc-effector functions through engagement with Fc receptors, IgG bound to MARV VLP (VLPs expressing MARV trimeric GP on surface of particles made of MARV matrix proteins VP40 and NP)^25^, was evaluated for binding to various FcɣRs by bead-based Luminex assay. No FcɣR binding was observed for the 10 uninfected control plasma samples. At 12 months post-MARV infection, plasma IgG bound to MARV VLP demonstrated good binding to FcɣRI and FcɣRIIA followed by FcɣRIIIA and FcɣRIIB, and no binding to FcɣRIIIB (Fig. 5). At 60 months post-infection, the binding of MARV VLP-specific IgG to FcɣRI and FcɣRIIIA remained stable. However, there was a gradual decline in binding to FcɣRIIA from 12 months to 60 months in these MVD survivors.

In summary, this collection of data demonstrates that MARV infection induced a diverse, long-lasting, non-neutralizing IgG with moderate affinity IgG antibody repertoire against MARV VLP and several proteins including GP. IgG-Fc-receptor binding activity to FcɣRI and FcɣRIIIA was also maintained for 5 years in this cohort of MVD survivors.

### Identification of immunodominant MARV antigenic sites to generate MARV binding and neutralizing antibodies as potential vaccine and therapeutic targets against MARV

**Fig. 6:**
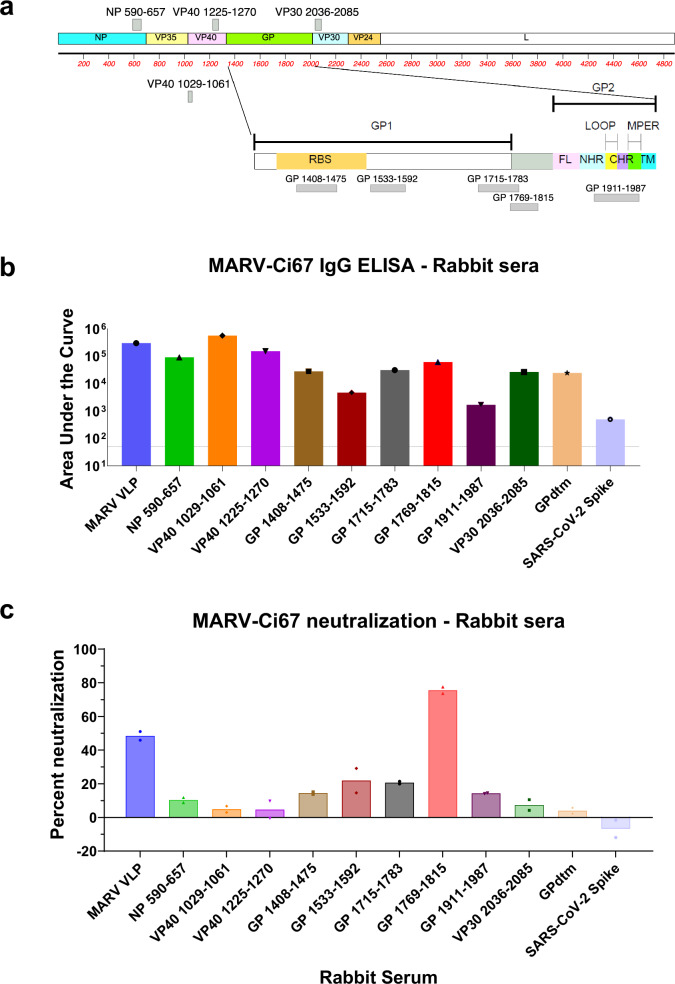
MARV-Ci67 binding and neutralization by serum antibodies generated following rabbit immunization with GFPDL selected immunodominant antigenic site peptides. **a** Elucidation of immunodominant antigenic sites against the MARV proteome following MARV infection in survivors. Immunodominant antigenic sites within the MARV proteome recognized by serum antibodies in MVD survivors (based on data presented in Fig. 2) are shown in gray bars aligned to the corresponding MARV protein sequence either above or below the proteome schematic. The antigenic sites discovered in MARV-GP using the post-infection antibodies are depicted below the MARV-GP schematic. **b** Serial dilution of serum samples obtained from rabbits immunized thrice with protein or KLH conjugated antigenic site peptides were analyzed for total IgG binding to MARV-Ci67 in ELISA. The area under the curve data shown is the mean of two rabbits per immunogen. **c** Virus neutralization titers were determined using wild-type MARV-Ci67 and BSL-4 based microneutralization assay. The average percent neutralization of a 1:10 dilution of sera of two rabbits per immunogen from duplicate assays is shown. Source data are provided as a Source Data file.

Currently, there is no approved or licensed vaccine against Marburg virus and most MARV vaccines under development generate low to no neutralizing antibody response in animal models. Moreover, antigenic domains within the MARV proteins that can be used as an immunogen to generate neutralizing antibodies against MARV are not clearly defined. To follow-up on GFPDL based human epitope profiling of MVD survivors and to determine the functional importance of the MARV-GFPDL proteome-wide data, we next investigated the immunogenic potential of GFPDL-identified immunodominant antigenic sites across the MARV proteome (Fig. 6a) to generate MARV binding and neutralizing antibodies that could potentially be used as vaccine or therapeutic targets. In addition, we included MARV VLPs (VLPs expressing trimeric GP on surface of particles made of MARV matrix proteins VP40 and NP) and recombinant MARV GP, which are targets for vaccine and therapeutic development against MARV. Rabbits were immunized with MARV VLPs, recombinant MARV GP (GPdtm), or nine individual KLH-conjugated synthetic peptides representing most of the immunodominant antigenic sites up to 70 amino acid residues long across MARV proteome (Fig. 6a).

Immunization of rabbits with all MARV antigenic site peptides, either from GP, NP, VP40, or VP30, generated binding antibodies against MARV-Ci67 virion particles as determined by ELISA, with the highest IgG binding observed for serum induced by VP40 peptides (Fig. 6b). To evaluate the neutralization activity of the rabbit immune sera, a microneutralization assay was performed with wild-type MARV-Ci67 in BSL-4. Immunization with MARV VLP elicited appreciable neutralizing antibodies, well above the neutralizing activity of negative control serum from SARS-CoV-2 spike immunized rabbits (Fig. 6c). Surprisingly, the rabbits immunized with recombinant MARV-GPdTM protein generated virus-binding antibodies, but these antibodies did not neutralize the MARV-Ci67. Neutralizing activity was generally associated with GP peptide immunization, with the highest neutralizing activity observed with GP 1769-1815 peptide, a neutralizing epitope in the wing domain of MARV GP (Fig. 6c). Peptide GP 1769-1815 sequence resides in the immature GP0 that is present in the peptide region that is cleaved off by proteases during maturation to generate mature GP1 and GP2.

These rabbit studies confirmed that GFPDL-identified antigenic peptides are immunogenic as they can elicit antibodies that bind MARV-Ci67. However, most of these binding antibodies are non-neutralizing, even the ones raised by immunizing the MARV-GP without the transmembrane domain. Importantly, rabbit immunization studies identified an immunodominant immunogenic site in the wing region between GP1 and GP2, that is only present in uncleaved GP0, generated the strongest neutralizing antibodies and is potentially a promising target for development of vaccines and therapeutics against MARV.

## Discussion

Marburg virus is a highly pathogenic virus, however, there are no approved vaccines or therapeutics against MARV, and it remains a threat to public health requiring urgent research and development of effective medical countermeasures. An in-depth understanding of the humoral immune response following MARV infection can facilitate knowledge-based development and evaluation of effective vaccines and antibody-based therapeutics against Marburg virus.

**Fig. 7:**
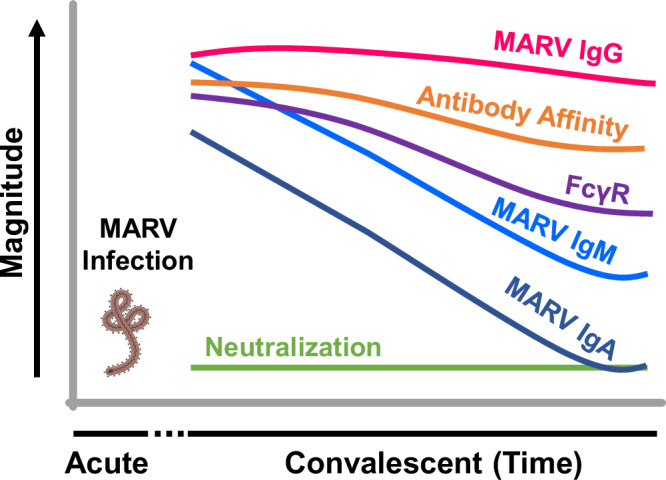
Longitudinal evolution of different antibody parameters against MARV following infection in MVD survivors over years. Schematic displaying the differential evolution of virion-binding IgG in ELISA and MARV neutralization titers, MARV specific IgM vs IgG vs IgA, antibody affinity maturation and Fc-receptor interaction across MARV proteome during convalescent phase over years in MVD survivors. The study data only portrays the antibody kinetics in the convalescent phase (post 12-months) in these MVD survivors.

Previous assessments of immune responses following human MARV infection have investigated a single cross-sectional time point for T cells and antibodies by ELISA and neutralization tests, which only provides one snapshot of the antibody response^11^. To our knowledge, this study represents the most comprehensive longitudinal characterization of durability, diversity, and quality of the polyclonal antibody repertoire across the complete MARV proteome and its evolution in humans, who survived MARV infections with supportive care alone without experimental therapies. The findings were very similar across the MVD survivor cohort. MARV neutralizing titers, SPR binding antibodies, and antibody affinity maturation to each of the MARV proteins, as well as Fc-receptor engagement, was very similar in 9 of the 10 MARV survivors, with one survivor showing lower Fc-receptor interaction than the rest of the group. Altogether, we observed a differentially evolving diverse but non-neutralizing antibody response to the MARV proteome over a five-year period in a cohort of MVD survivors (Fig. 7). A time-dependent decline in IgM and faster decay in IgA binding antibodies against the MARV proteome is observed from 12-months to 60-months post-exposure. MARV-specific IgG titer and antibody affinity is maintained during the convalescent phase and demonstrate good binding to various activating FcɣRs involved in ADCP and ADCC functions.

The MARV-proteome wide antibody profiling showed a diverse but differential evolution of IgM vs IgG vs IgA antibody epitope repertoire, however, only the IgG epitope repertoire persisted for up to 5 years post-exposure. The highest antibody reactivity, antibody epitope diversity, and antibody affinity were observed against the most abundant matrix protein VP40, followed by GP, VP35, and VP24, then NP, and minimal against VP30 and L polymerase. The differential antibody kinetics to various MARV proteins suggests disparate expression and/or antigen exposure/recognition by the human immune system following MARV infection in these MVD survivors. This contrasts with our previous study with Ebola virus, wherein a long-lasting durable IgM response was observed following EBOV infection up to more than 2 years that was comparable or higher than the IgG repertoire^17^. In contrast, in this longitudinal study, MARV infection-induced immune response in MVD survivors shows a decline of IgM and IgA in comparison with durable IgG repertoire. Both MARV and EBOV are known to persist in immune-privileged sites (testicles, eye, placenta, amniotic fluid, etc.) in some survivors who have recovered from Marburg virus disease or Ebola virus disease, respectively^3–7^. So, the dynamics of IgG vs IgA vs IgM repertoire in the current study following MARV infection were unexpected and are different from the EBOV induced IgM repertoire, a related filovirus. This could possibly be due to differences in the persistence/distribution of EBOV vs MARV in the human body, and its dynamics of interaction with a human immune system that results in differential durability of IgM repertoire against various MARV proteins at different time-points in the MVD survivors. All 10 MVD survivors were negative for MARV RNA in blood at time of sample collection by RT-qPCR. One of the possible limitations of GFPDL-based assessments is that while the phage display is likely to detect both conformational and linear epitopes on MARV proteins, they are unlikely to detect paratopic interactions that require post-translational modifications and rare quaternary epitopes that cross-protomers. However, in the current study (Supplementary Fig. S2) and prior studies with post-infection or post-vaccination human polyclonal antibodies against other viruses, >90% of antibodies were removed by adsorption with the GFPDL, supporting the use of the GFPDL for analyzes of human plasma^13–15,17,26–31^.

Surprisingly, MARV infection is one of the very few viruses that does not generate durable neutralizing antibody response in humans following infection, especially in comparison to another filovirus like Ebola. Therefore, for the first time in humans, we evaluated non-neutralizing FcR interactions following MARV infection that may play a role in disease perturbation and the development of vaccines and therapeutics. Cell-based FcR assays require target and effector cells that may give variabilities in results depending on the conditions of the cells. Due to the limitation of sample volumes, measuring antibody binding level of FcɣRs by bead-based assay provide robust results and reduce time and resources compared to cell-based assays. In earlier studies, the bead-based read-out of IgG-Fc binding to FcɣR strongly correlated to Fc-mediated functions like ADCC and ADCP^21,32^. Each FcɣR is involved in a specific effector function such that FcɣRI and FcɣRIIA are responsible for ADCP whereas FcɣRIIIA is involved in ADCC^21^. Despite the anti-GP IgGs being poorly neutralizing, they showed good binding to activating FcɣRI, FcɣRIIA and FcɣRIIIA receptors that persisted for up to 5 years in these MVD survivors. The higher binding to FcɣRI and FcɣRIIA rather than FcɣRIIIA, suggests that post-infectious convalescent plasma may have higher ADCP activities than ADCC. The FcR functions of antibody can play an important role in disease perturbation, as was observed previously for non-neutralizing MAb MR228 showing ADCP and NK cell activation but not ADCC against MARV^33^. Durability of anti-GP IgG with strong FcR binding activity in these MVD survivors suggests that it should be feasible to generate long-lasting anti-GP IgG as well as memory B-cells by different vaccine approaches including vectored, protein, DNA, and mRNA-based vaccines in humans. Some of these approaches are in early clinical trials that are measuring GP-binding antibodies to evaluate vaccine immunogenicity^34^. Even in the absence of durable neutralization activity, analyzing the Fc-receptor interactions in the vaccine clinical trials would be important to define a Fc-mediated function as a correlate of protection against MVD.

Neutralizing antibodies targeting GPs of filoviruses are known to provide therapeutic protection against both Ebolavirus and Marburgvirus^35–41^ and in turn, eliciting neutralizing antibody titers has been a desirable objective of vaccine development efforts. Short-lived neutralizing titers in MVD survivors may be related to relatively few neutralizing epitopes within MARV GP compared with GPs of Ebolaviruses. To date, the only known neutralizing epitopes of MARV GP are located at or near the RBD^35^. In contrast, several neutralizing epitopes in the RBD, internal fusion loop (IFL), and stalk/MPER, and to a lesser degree in the glycan cap, have been identified for Ebolavirus GPs^42,43^. This may be due to the purported location of the mucin domain of MARV GP being more equatorial relative to the viral membrane, which likely occludes epitopes of the IFL, base and stalk, whereas the mucin domain of Ebolaviruses is in a more upward position, leaving these same epitopes more exposed^44^. The combination of fewer neutralizing epitopes within MARV GP and MARV-specific RBD binding antibodies being relatively less potent neutralizers compared to the neutralizing potency of EBOV-specific IFL and base binding antibodies may explain the rapid decline in neutralizing titers observed for the cohort of MVD survivors evaluated in this study. However, these findings are limited to convalescent phase in these 10 MVD survivors that should be further expanded to in-depth longitudinal analyzes of acute MARV infection to convalescence in a larger cohort of MVD survivors.

Most MARV vaccines currently under development generate low to no neutralizing antibody response in animal models. In fact, vaccine-elicited neutralizing antibody titers were not correlated with vaccine efficacy in some previous studies. Vaccination of nonhuman primates with a recombinant Vesicular Stomatitis Virus vector vaccine expressing the glycoprotein of MARV elicited high levels of GP-specific IgG with poor neutralizing activity, yet was completely protective against MARV challenge^45,46^. Interestingly, in a durability study with this same vaccine, MARV GP-specific IgG titers remained relatively constant over a 400-day period while neutralizing titers waned during that same period and remained relatively low^47^. Despite having low neutralizing antibody titers, all vaccinated NHPs survived exposure to MARV when challenge 400 days post-vaccination, though neutralizing antibody titers were significantly higher by day 28 post-exposure, suggesting MARV infection-induced immune response. Domains or antigenic sites that can be used as an immunogen to generate neutralizing antibodies against MARV are not clearly defined for MARV proteins. To identify potential neutralization targets for vaccine and therapeutic development, we followed-up on our comprehensive GFPDL-based human serum epitope profiling of MVD survivors. Therefore, rabbit immunization studies were performed to define the immunogenic potential of GFPDL-identified immunodominant antigenic sites and the functional importance of the antibodies generated by these immunogens as potential vaccine or therapeutics targets against MARV. All rabbits immunized with MARV proteins/peptides generated antibodies that bound strongly to MARV-Ci67 particles. Interestingly, only GP presented in the context of a VLP, but not recombinant GP without transmembrane domain, generated neutralized antibodies, suggesting a role of spatial presentation of GP to induce neutralizing antibody response. Although all peptides elicited MARV-Ci67 reactive antibodies, only antibodies against antigenic site peptide GP 1769-1815, located within the highly conserved wing domain of MARV GP, appreciably neutralized wild type MARV-Ci67. Interestingly, this small protein domain encompassing the conserved wing region between GP1 and GP2 induced neutralizing antibodies against MARV, when used alone as protein domain, but not when this domain sequence is present as a part of the entire GP ectodomain (Fig. 6c). Previously MAbs targeting the wing domain showed weak neutralizing activity, although they were protective in mice and, to a lesser degree, in guinea pigs^33,48^. Like many non-neutralizing antibodies, the protective efficacy of these wing binding MAbs was determined to be dependent on Fc effector function. Unfortunately, due to the COVID-19 pandemic and limited BSL-4 capacity, corresponding MARV vaccine efficacy studies have not yet been performed. This study demonstrates that in-depth longitudinal antibody profiling of the host-MARV interaction identified an immunogen in the conserved wing region between GP1 and GP2 that induced neutralizing antibodies against MARV.

In summary, MARV infection generates a diverse, long lasting, moderate affinity IgG antibody repertoire that may play a role in disease perturbation using FcɣR activity. Since MARV-induced neutralizing antibody responses are rather short lived in MVD survivors, vaccine platforms that can generate robust cell-mediated immunity or antibody Fc-mediated effector functions may provide protection from MVD. In fact, non-neutralizing, Fc effector function modulating MARV MAbs are known to be protective on their own and can also enhance potency of RBD binding MAbs when used in combination, further validating the importance of non-neutralizing MARV-specific antibodies^33^. Antibodies targeting the wing domain may play in important role in protection against MARV and an immunogen designed based on the wing domain of MARV-GP can be promising vaccine against MARV. The comprehensive longitudinal immune profiling provides a more in-depth understanding of quantitative and qualitative aspects of MARV antibody profile. This could aid the development and evaluation of more effective targeted therapeutic and vaccine approaches that could influence the transmission and clinical outcome against Marburg virus.

## Methods

### Proteins and clinical samples

Recombinant Marburg virus-like particles (VLPs expressing MARV trimeric GP on surface of particles made of MARV matrix proteins VP40 and NP)^25^, and MARV GP, NP, VP35, VP40, VP24, VP30 and L polymerase proteins were purchased from MyBioSource, IBT Bioservices, and Creative Diagnostics Inc.

The longitudinal post-MARV infection convalescent clinical plasma samples and matched uninfected controls locally from Uganda were obtained during months and years that followed the 2012 outbreak in Uganda. The human use protocols for collecting samples from MVD survivors in Uganda were reviewed and approved by the Helsinki committees of the Uganda Virus Research Institute in Entebbe, Uganda (reference number GC/127/13/01/15) and the Ugandan National Council for Science and Technology (registration number HS1332). Participants included confirmed MVD survivors, according to patient PCR and ELISA results, from the MARV outbreak of 2012 in the Ibanda and Kabale districts of Uganda, as well as healthy local community members that were not infected. Survivors in these studies ranged in age from 18 to 54 years. Health questionnaires administered before sample collection indicated that the study participants were relatively healthy, with a few individuals reporting nonspecific malaise. One survivor was receiving medication for malaria. Plasma was purified from whole blood samples collected during convalescence ( ~ 9 months post-recovery) from 12 to 60 months post-infection and all blood specimens from these individuals were negative for MARV RNA at time of collection.

A positive control post-MARV infection human serum sample with known neutralization activity against MARV was included in all the PRNT assays. There wasn’t much of this serum left, so we were not able to include this sample in any of the subsequent antibody assays.

Samples were tested in different antibody assays with approval from the U.S. Food and Drug Administration’s Research Involving Human Subjects Committee (FDA-RIHSC) under exemption protocol #15-064B.

#### Ethics statement for human study

Written informed consent as well as a personal health questionnaire was completed for each participant during each collection timepoint. The study at CBER, FDA was conducted with de-identified samples under Research Involving Human Subjects (RIHSC) exemption #15-064B; and all assays performed fell within the permissible usages in the original consent. As described previously^11^, human use protocols for collecting samples from MVD survivors in Uganda were reviewed and approved by the Helsinki committees of the Uganda Virus Research Institute in Entebbe, Uganda (reference number GC/127/13/01/15) and the Ugandan National Council for Science and Technology (registration number HS1332).

### Marburg virus plaque reduction neutralization test (PRNT)

PRNTs were performed as previously described^49^ with slight modifications. Ten-fold serial dilutions of plasma were mixed with 100 plaque-forming units (pfu) of Marburg virus-Ci67 at 37 °C for 1 h in the presence of 5% guinea pig complement (Cedarlane). Plasma/virus mixture was used to infect Vero cell monolayers for 1 h at 37 °C, after which, cells were overlaid with agarose and incubated at 37 °C. A second overlay containing 5% neutral red was added 6 days later and plaques were counted the next day. Neutralization titers were determined to be the last dilution of plasma that reduced the number of plaques by 50% compared with control wells. A positive control human serum sample with known neutralization activity against MARV with a PRNT50 titer of 1:160 was included in all the neutralization assays that demonstrated consistent neutralization titers across all assays. Plaque reduction neutralization assay is highly reproductible and have been widely used for multiple humans and NHP studies and were performed in the BSL-4 laboratory of the United States Army Medical Research Institute of Infectious Diseases.

### MARV gene fragment phage display library (GFPDL) construction

The cDNA complementary to all the ORFs of MARV was chemically synthesized and used for cloning. Purified DNA containing each MARV ORF were combined at equimolar amounts and then digested with *DNase I* to obtain gene fragments of 50–1000 bp size range, and used for GFPDL construction as described previously^13,14,17^. A gIII display-based phage vector, fSK-9-3, was used where the desired polypeptide can be displayed on the surface of the phage as a gIII-fusion protein. The phage libraries were constructed from the whole MARV genome, potentially display viral protein segments ranging in size from 15 to 350 amino acids from the entire MARV proteome, as fusion protein on the surface of bacteriophage (Supplementary Fig. 1).

### Affinity selection of MARV GFPDL phages with polyclonal human plasma

Prior to panning of GFPDL with polyclonal plasma antibodies, plasma components that could non-specifically interact with phage proteins were removed by incubation with UV-killed M13K07 phage-coated Petri dishes. Equal volumes of each human plasma of 10 survivors were pooled and used for GFPDL panning. GFPDL affinity selection was carried out in-solution with anti-IgM, or protein A/G (IgG), or anti-IgA specific affinity resin as previously described^13–15,17,28^. Briefly, the pooled plasma was incubated with the GFPDL and the specific resin, the unbound phages were removed by PBST (PBS containing 0.1 % Tween-20) wash followed by washes with PBS. Bound phages were eluted by addition of 0.1 N glycine-HCl pH 2.2 and neutralized by adding 8 µL of 2 M Tris solution per 100 µl eluate. After panning, antibody-bound phage clones were amplified, the inserts were sequenced, and the translated sequences were aligned to the MARV proteome, to define the epitope specificity in these MVD survivors. The GFPDL affinity selection was performed in duplicate (two independent experiments by a research fellow in the laboratory who was blinded to sample identity), and similar number of phage clones and epitope repertoire observed in both phage display analyses.

### Adsorption of polyclonal human post-infection plasma on MARV GFPDL phages and residual reactivity to MARV-GP

Prior to the panning of GFPDL, 500 μL of 10-fold diluted plasma antibodies from post-infection sample was adsorbed by incubation with MARV GFPDL phage-coated Petri dishes. To ascertain the residual antibodies specificity, an ELISA was performed with wells coated with 100 ng/100 μL of recombinant MARV-GP. After blocking with PBST containing 5% milk, serial dilutions of human plasma (with or without GFPDL adsorption) in blocking solution were added to each well, incubated for 1 hr at RT, followed by addition of 5000-fold diluted HRP-conjugated donkey anti-human IgG-Fc specific antibody and developed by 100 μL of OPD substrate solution. Absorbance was measured at 490 nm.

### Antibody binding kinetics of post-MARV infection plasma to recombinant MARV proteins by Surface Plasmon Resonance (SPR)

Steady-state equilibrium binding of post-infection human polyclonal sample was monitored at 25 °C using a ProteOn surface plasmon resonance (BioRad). The purified recombinant MARV proteins were captured to a Ni-NTA sensor chip or on the GLC sensor ship with 200 resonance units (RU) in the test flow channels. The protein density on the chip was optimized such as to measure monovalent interactions independent of the antibody isotype. Serial dilutions (10-, 30- and 90-fold) of freshly prepared samples in BSA-PBST buffer (PBS pH 7.4 buffer with Tween-20 and BSA) were injected at a flow rate of 50 µL/min (120 sec contact duration) for association, and disassociation was performed over a 600-second interval. Responses from the protein surface were corrected for the response from a mock surface and for responses from a buffer-only injection. SPR was performed with serially diluted sample of each individual timepoint in this study. Total antibody binding analysis were calculated with BioRad ProteOn manager software (version 3.1). All SPR experiments were performed twice and the researchers performing the assay were blinded to sample identity. In these optimized SPR conditions, the variation for each sample in duplicate SPR runs was <7%. The maximum resonance units (Max RU) data shown in the figures is for the 10-fold diluted sample.

Antibody off-rate constants, which describe the stability of the antigen-antibody complex, i.e., the fraction of complexes that decays per second, were determined directly from the human polyclonal sample interaction with recombinant MARV proteins using SPR. To that end, serially diluted plasma at 10-, 30- and 90-fold dilutions were analyzed to determine antibody off-rate constants, which describe the fraction of antigen-antibody complexes that decay per second in the dissociation phase, only for the sensorgrams with maximum RU in range of 10-100 RU (Supplementary Fig. S7) and calculated using the BioRad ProteOn manager software for the heterogeneous sample model.

### Fcγ-receptor binding assay

MARV VLP and a human serum albumin protein were conjugated to magnetic beads that contains different region numbers using Bio-Plex amine coupling kit (Bio-Rad), as per manufacturer’s interactions. Coupled beads were mixed in assay buffer (1% BSA/1X PBS-Tween 20) to have 500 beads per protein in 50 µL/well in 96-well flat bottom plates. Plates were washed with Bio-Plex wash buffer using Bio-Plex Pro wash station, and plasma samples were added to each well in 10-fold dilution. Beads coupled to human serum albumin were included as a control. Plates were incubated on a microplate shaker at 850 rpm for 1 hr at room temperature. After incubation, plates were washed and biotinylated human FcγR proteins were added. Plates were incubated for 30 min at room temperature on a microplate shaker at 850 rpm followed by washing with Bio-Plex wash buffer. Bio-Plex Streptavidin-PE was added and incubated on a microplate shaker at 850 rpm for 30 min at room temperature. Plates were washed again and 125 µL of assay buffer was added to each well. Data was acquired using Bio-Plex 200 system. Each sample was run in duplicate, and all data were normalized by subtracting values of human serum albumin.

### Peptide fragment conjugation to KLH carrier protein

The peptide conjugation to Maleimide Activated KLH was performed as described in the product manual of Imject® Maleimide Activated KLH (Product 77605, Thermo Scientific).

### Rabbit immunization studies

Female 4 to 6-weeks old New Zealand white rabbits (two per immunogen) were immunized thrice intra-muscularly at 21-days interval with 50 mcg of VLP or recombinant GP-deltaTM protein or SARS-CoV-2 spike (control) or 20 μg of KLH-conjugated peptides mixed with Emulsigen Adjuvant. Sera were collected before (pre-vaccination) and after 3^rd^ vaccination and analyzed for binding antibodies in ELISA and neutralization assay against MARV Ci67. The rabbit study was carried out in strict accordance with the recommendations in the Guide for the Care and Use of Laboratory Animals of the National Institutes of Health and were performed under BSL2 conditions.

### Ethics statement for rabbit study

The rabbit experiments were approved by the U.S. FDA Institutional Animal Care and Use Committee (IACUC) under Protocol #2008-10. The animal care and use protocol meets National Institutes of Health guidelines.

### Marburg virion IgG enzyme-linked immunosorbent assay (ELISA)

MARV-specific IgG titers were determined by ELISA as previously described^10^ with slight modifications. Briefly, polyvinyl chloride microtiter plates (Dynatech Laboratories) were coated with irradiated MARV-Ci67, diluted in phosphate-buffered saline (PBS), and incubated overnight at 4 °C before blocking with 5% milk protein in PBS/0.02% Tween-20 at room temperature. Rabbit immune serum samples were serially diluted in blocking buffer and added to antigen-coated plates for 2 hours (h) at room temperature. Plates were washed with wash buffer (PBS/0.02% Tween-20) before adding 1:5000 dilution of horseradish peroxidase (HRP)- conjugated goat anti-rabbit Fc fragment specific IgG (Jackson Immuno Research) for 1 h at room temperature. Following a final wash, 2,2′-Azinobis [3-ethylbenzothiazoline-6-sulfonic acid]-diammonium salt (ABTS) substrate (Kirkegaard and Perry Laboratories, Inc.) was added, and absorbance values were read at 405 nm using a Spectramax plate reader after 30 min (Molecular Devices, LLC). Cutoff values for each dilution were calculated using absorbance values of naïve plasma for that dilution and the following formula: average absorbance + 3 SD. End-point titers for each plasma sample are expressed as the last dilution to exceed the cutoff value for a given dilution.

### Marburg virus microneutralization assay

Rabbit serum samples were assessed for neutralization activity against MARV Ci67 by microneutralization assay. Ninety-six well black plates (Griener Bio-One) were seeded with ATCC Vero E6 cells and incubated overnight at 37 °C. The next day, heat inactivated (56 °C for 30 min) serum samples were diluted 1:10 in media and taken into BSL-4 where MARV Ci67 was added to the serum at an MOI of 0.4 (1 × 10^4^ pfu) and incubated for 1 h at 37 °C. The antibody/virus mixture was added to the cells and incubated for 1 h at 37 °C. The virus/antibody mixture was decanted, cells were washed with PBS (Sigma), media was added back to the cells, and plates were incubated for 48 h at 37 °C before inactivating with 10% formalin (Valtech). Inactivated plates were then brought out of BSL-4 and blocked with cell stain buffer (BioLegend) and incubated for 2 h with a MARV specific antibody 7E6 (USAMRIID). Plates were washed 3 times with PBS and stained with 1:2000 dilution of AlexaFluor 488 conjugated goat anti-mouse antibody (Invitrogen) for 1 h at RT. Plates were washed and Hoechst stain (Invitrogen) was added before imaging on a Bio Tek Cytation where percent infection was determined by AlexFluor 488 positive cells divided by total number of nuclei.

### Statistical analysis

The half-life (T1/2) of polyclonal binding antibodies for antibody decay against various MARV proteins in the convalescent phase in these MVD survivors was calculated using the nonlinear regression one-phase decay model in GraphPad Prism 9.3.1 (GraphPad Software).

### Reporting summary

Further information on research design is available in the Nature Portfolio Reporting Summary linked to this article.

## Supplementary information

Supplementary Information

Peer Review File

Reporting Summary

## Source data

Source Data

## Data Availability

All data are shown in the manuscript figures and supplementary information. Source data are provided with this paper. Both the GFPDL technology and data are patented and can be shared upon appropriate agreements with FDA technology transfer office, as required for any patented invention. The GFPDL technology described is covered by US and international patents. GFPDL technology can be made available under licensing of technology from FDA technology transfer office. The GFPDL sequence data is shown in Fig. 2, Supplementary Figs. S1, S4, S5, and S6 described in this study can be made available upon request from academic and government scientists under MTA for non-commercial usage of these datasets. The sequencing data can be made available on request from the corresponding author (S.K.) upon signing of appropriate agreements and can be provided within 2-4 weeks of signing the appropriate agreement. For commercial use, the license can be obtained from FDA technology transfer office. Source data are provided with this paper.

## Code availability

No custom codes were used in this study.
