# Supplementary material for: Longitudinal proteome-wide antibody profiling in Marburg virus survivors identifies wing domain immunogen for vaccine design
Source: Nat Commun. 2024 Sep 17;15:8133. doi: 10.1038/s41467-024-51021-5 (PMC11405854; doi:10.1038/s41467-024-51021-5)
Supplement: Supplementary file 1 — Supplementary Information [file 41467_2024_51021_MOESM1_ESM.pdf]

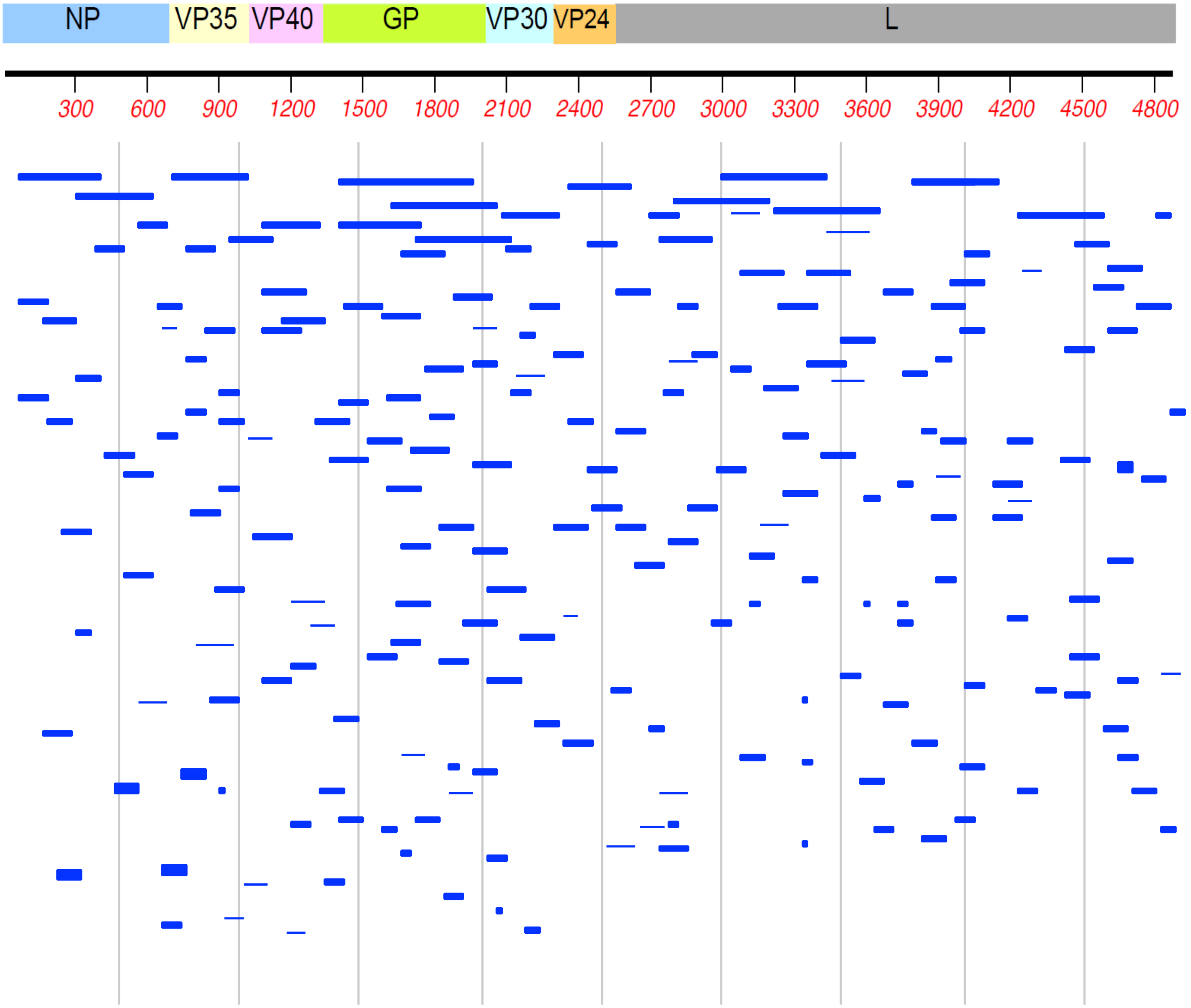

Supplementary Figure S1. Random distribution of size and sequence of the MARV-GFPDL. Sequencing of Marburg virus proteome sequences expressed by the phages of the MARV GFPD libraries were aligned to the Marburg virus proteome translated sequence.

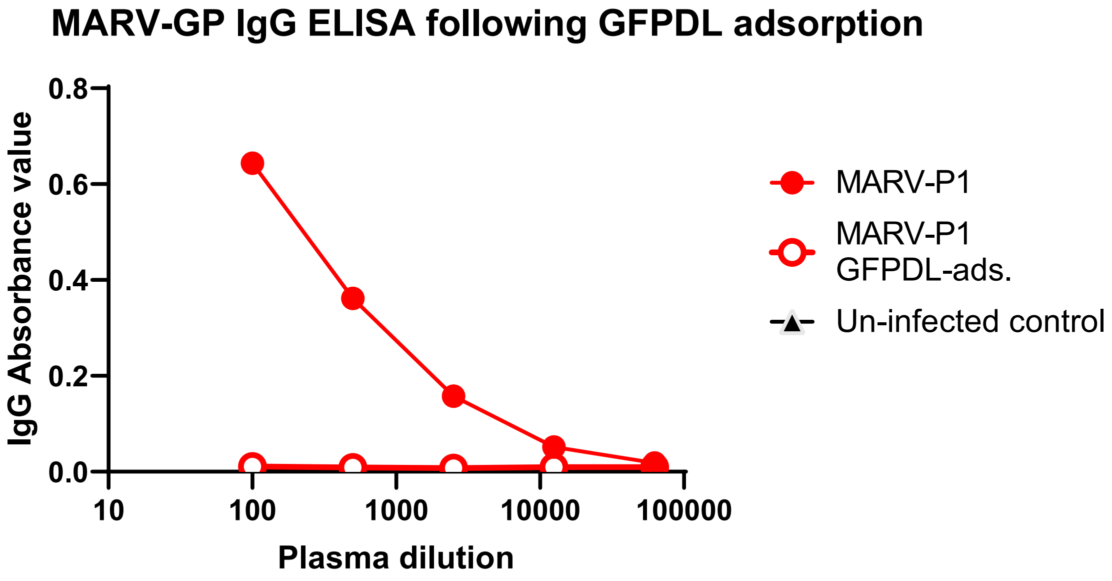

Supplementary Figure S2. MARV anti-GP reactivity of MARV convalescent plasma in ELISA before and after MARV-GFPDL adsorption. Post-MARV infected MVD survivor's plasma (#P1) was adsorbed on MARV GFPDL coated petri dishes. Binding to recombinant MARV-GP is shown before (filled red symbol) and after (empty red symbol) GFPDL-adsorption or un-infected control (black symbol) in ELISA using HRP-conjugated donkey anti-human IgG-Fc specific antibody.

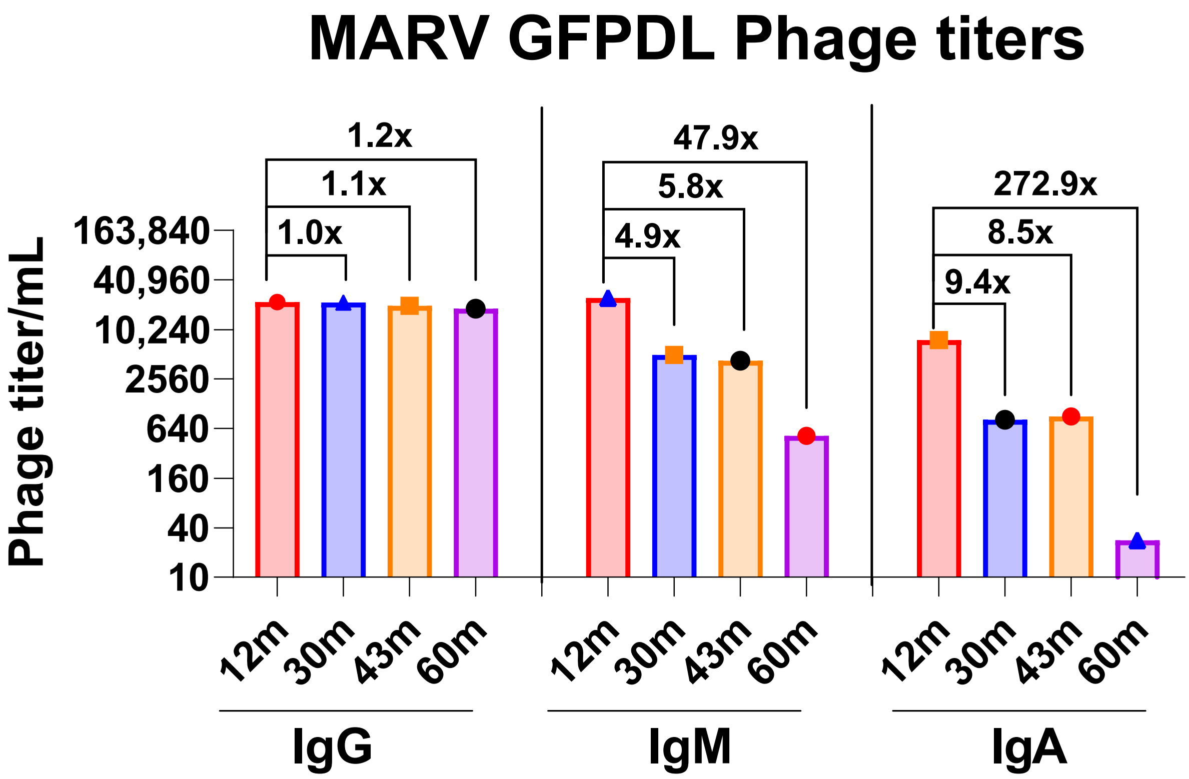

Supplementary Figure S3. GFPDL recognized phage titers by IgM, IgG and IgA antibodies in plasma of MVD survivors. Number of bound phage clones isolated using whole genome MARV GFPDL affinity selection on 12-, 30-, 43- or 60 months following MARV infection by IgG, IgM and IgA antibodies in MVD survivors.

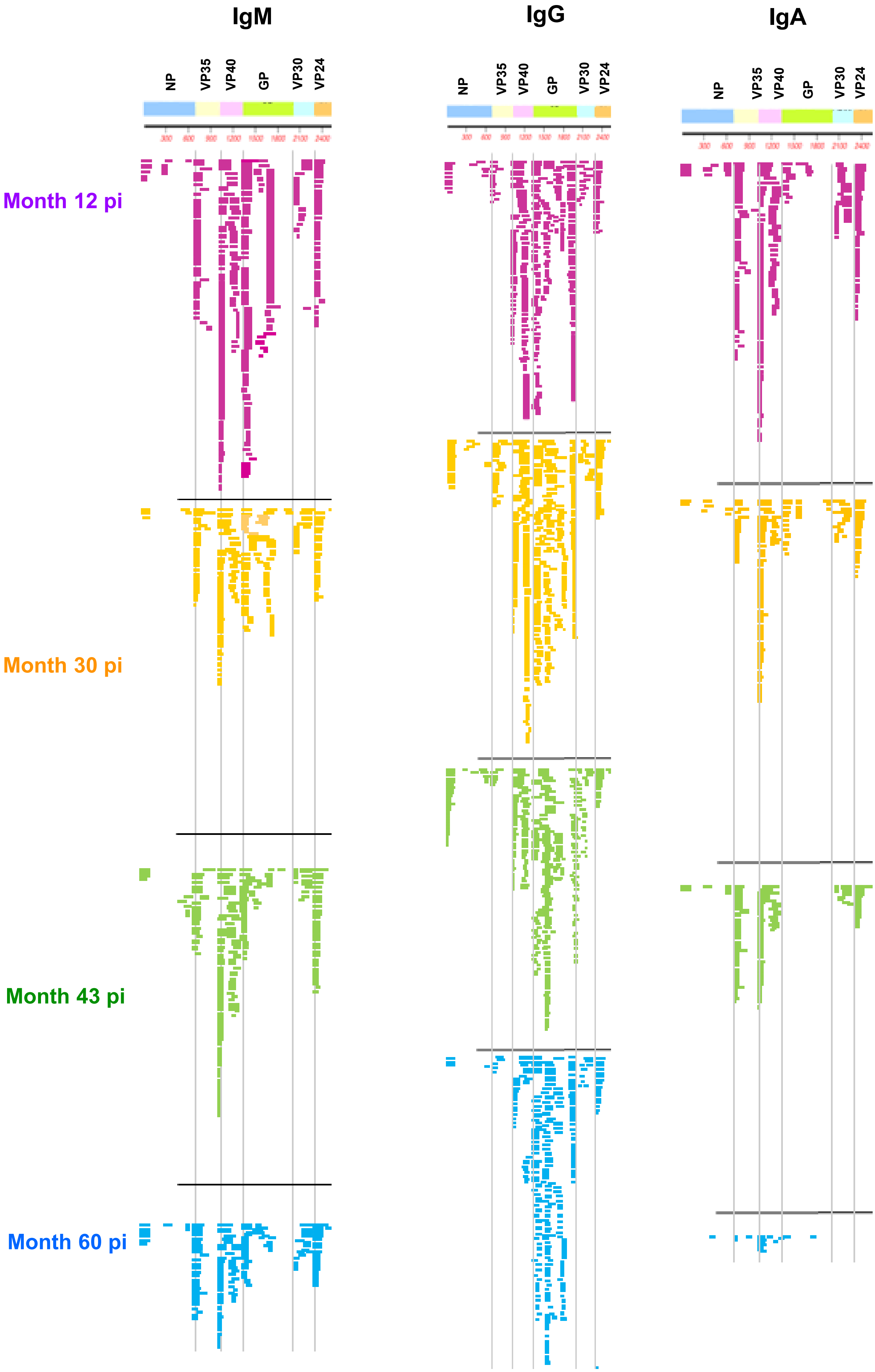
IgM
IgG
IgA
NP
VP35
VP40
GP
VP30
VP24
Month 12 pi
Month 30 pi
Month 43 pi
Month 60 pi

Supplementary Figure S4. Longitudinal analysis of MARV proteome-wide IgM, IgG, and IgA antibody epitope repertoires in MVD survivors. IgM, IgG, and IgA antibody epitope repertoire recognized in the human plasma at different months post-MARV infection (pi) during convalescence (12-, 30-, 43- and 60-months pi are colour coded) and their alignment to the whole proteome of MARV sans L polymerase (showing different proteins: NP, VP35, VP40, GP, VP30 and VP24). Graphical distribution of representative clones with a frequency of >2, obtained after affinity selection, are shown. No repetitive phage clones were identified in this immune inert protein L. The horizontal position and the length of the bars indicate the peptide sequence displayed on the selected phage clone to its homologous sequence in the MARV proteome on alignment. The thickness of each bar represents the frequency of repetitively isolated phage. The GFPDL affinity selection data was performed in duplicate (two independent experiments by researcher in the lab, who was blinded to sample identity), and similar number of phage clones and epitope repertoire was observed in both phage display analysis.

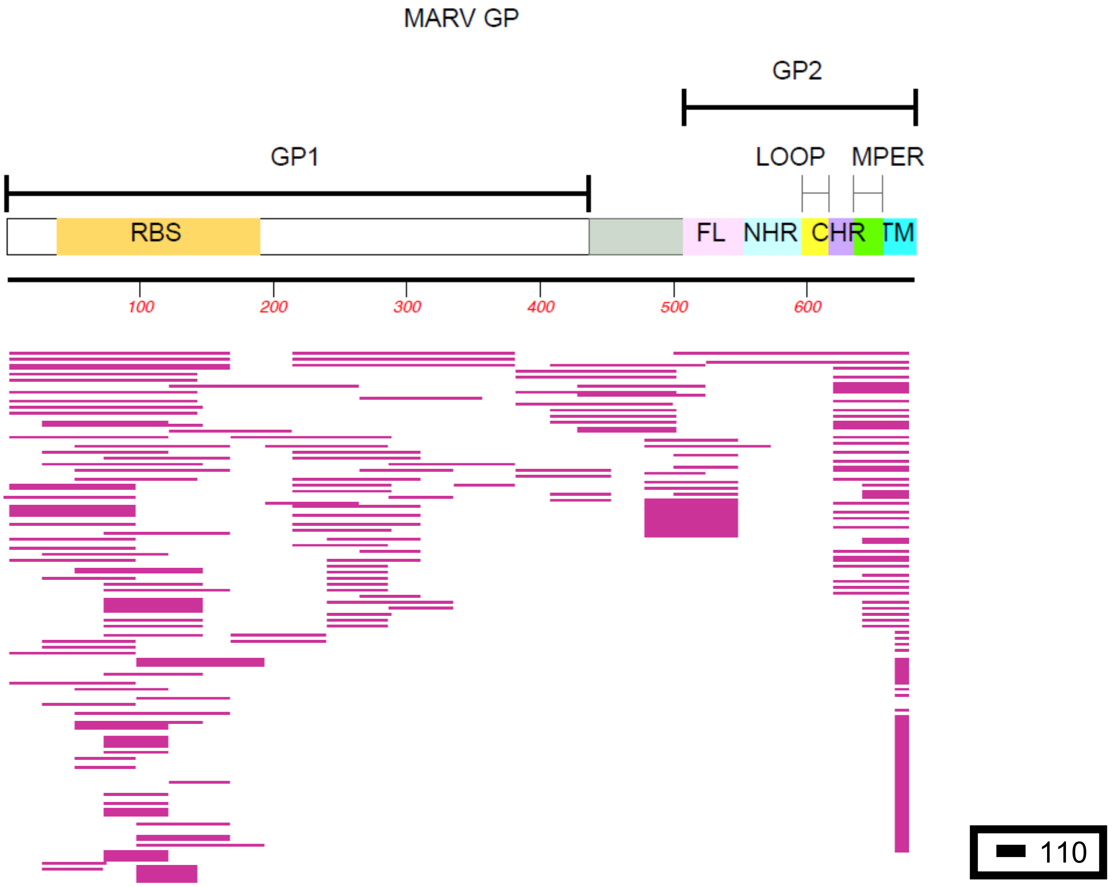

Supplementary Figure S5. MARV-GFPDL identified IgG epitope repertoire elicited against MARV GP in MVD survivors at 12-months post-exposure. IgG antibody epitope repertoire recognized in the 12-months post-infection plasma from MVD survivors across the MARV-GP. The schematic on top shows various domains in the GP of MARV: GP1, receptor binding site (RBS), GP2, fusion loop (FL), N-terminal heptad repeat (NHR), C-terminal heptad repeat (CHR), membrane proximal external region (MPER) and transmembrane (TM) domain. Graphical distribution of representative phage clones with a frequency of >3, obtained after IgG affinity selection are shown. The horizontal position and the length of the bars indicate the peptide sequence displayed on the selected phage clone to its homologous sequence in the MARV-GP on alignment. The thickness of each bar represents the frequency of repetitively isolated phage, with the scale shown below the alignment. . The GFPDL affinity selection data was performed in duplicate (two independent experiments by researcher in the lab, who was blinded to sample identity), and similar number of phage clones and epitope repertoire was observed in both phage display analysis.

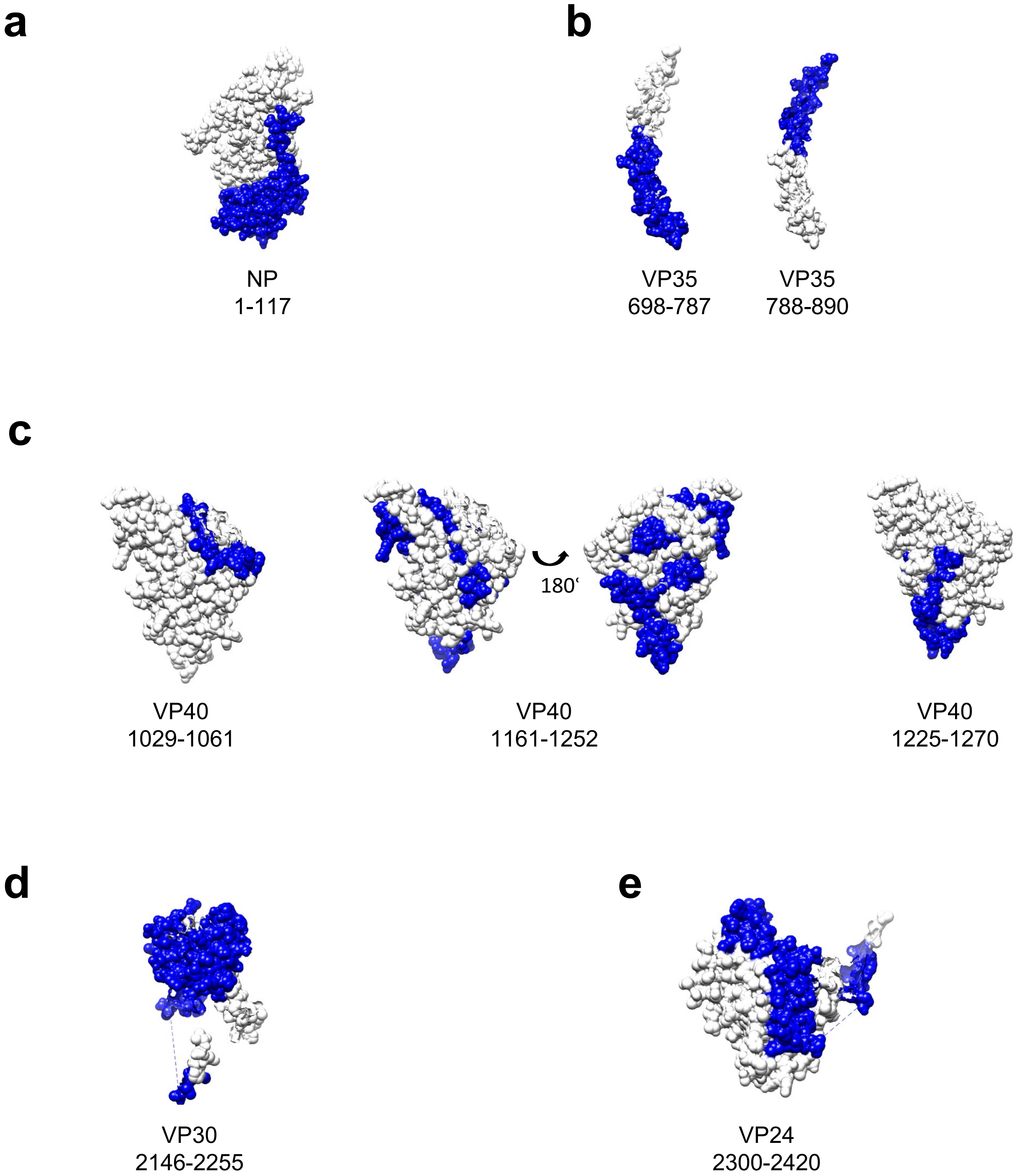

Supplementary Figure S6. Structural representations of antigenic sites on the surface structure of MARV proteins. GFPDL identified immunodominant antigenic sites recognized by IgG in the MVD survivors are depicted in blue on the structures of various MARV proteins (a) NP (PDB:7F1M), (b) VP35 (PDB:5TOH), and (c) VP40 (PDB:5B0V), (d) VP30 (PDB:5T3W) and (e) VP24 (PDB:4OR8). Residues depicted in blue are indicated for each structure correspond to the complete MARV proteome used for GFPDL. Several immunodominant sites could not be depicted since the structures were solved for the truncated MARV proteins.

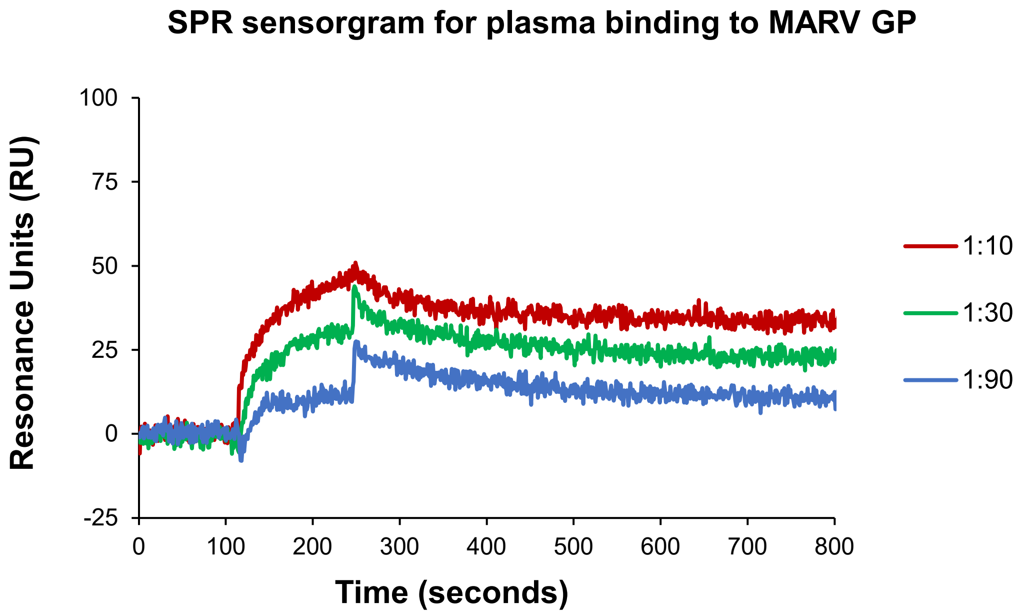

Supplementary Figure S7. Steady-state equilibrium analysis of plasma antibodies binding by SPR. Serial dilutions of plasma samples were injected simultaneously onto both MARV GP captured on a Ni-NTA sensor chip and on a surface free of protein (used as a blank). Binding responses from the protein surface were corrected for the response from the mock surface and for responses from a separate, buffer only injection. Uninfected control sample at 10-fold dilution did not show any binding in SPR. Antibody off-rate constants, which describe the fraction of antigen-antibody complexes that decay per second, were determined directly from the plasma sample interaction with GP using SPR in the dissociation phase only for the sensorgrams with Max RU in the range of 10-100 RU and calculated using the BioRad ProteOn manager software for the heterogeneous sample model.
