# Supplementary material for: Longitudinal proteome-wide antibody profiling in Marburg virus survivors identifies wing domain immunogen for vaccine design
Source: Nat Commun. 2024 Sep 17;15:8133. doi: 10.1038/s41467-024-51021-5 (PMC11405854; doi:10.1038/s41467-024-51021-5)
Supplement: Supplementary file 3 — Reporting Summary [file 41467_2024_51021_MOESM3_ESM.pdf]

nature portfolio

Corresponding author(s): Surender Khurana

Last updated by author(s): Jul 6, 2024

Reporting Summary

Nature Portfolio wishes to improve the reproducibility of the work that we publish. This form provides structure for consistency and transparency in reporting. For further information on Nature Portfolio policies, see our Editorial Policies and the Editorial Policy Checklist.

Statistics

For all statistical analyses, confirm that the following items are present in the figure legend, table legend, main text, or Methods section.

n/a	Confirmed	
☐	☒	The exact sample size (*n*) for each experimental group/condition, given as a discrete number and unit of measurement
☐	☒	A statement on whether measurements were taken from distinct samples or whether the same sample was measured repeatedly
☒	☐	The statistical test(s) used AND whether they are one- or two-sided<br>*Only common tests should be described solely by name; describe more complex techniques in the Methods section.*
☒	☐	A description of all covariates tested
☒	☐	A description of any assumptions or corrections, such as tests of normality and adjustment for multiple comparisons
☐	☒	A full description of the statistical parameters including central tendency (e.g. means) or other basic estimates (e.g. regression coefficient) AND variation (e.g. standard deviation) or associated estimates of uncertainty (e.g. confidence intervals)
☒	☐	For null hypothesis testing, the test statistic (e.g. *F*, *t*, *r*) with confidence intervals, effect sizes, degrees of freedom and *P* value noted<br>*Give P values as exact values whenever suitable.*
☒	☐	For Bayesian analysis, information on the choice of priors and Markov chain Monte Carlo settings
☒	☐	For hierarchical and complex designs, identification of the appropriate level for tests and full reporting of outcomes
☒	☐	Estimates of effect sizes (e.g. Cohen's *d*, Pearson's *r*), indicating how they were calculated

Our web collection on statistics for biologists contains articles on many of the points above.

Software and code

Policy information about availability of computer code

Data collection	Antibody data was collected in MS Excel version 16.57. BioRad ProteON Manager software (Version 3.1.0) was used to collect antibody binding data from SPR machine (www.Biorad.com)
Data analysis	BioRad ProteON Manager software (Version 3.1.0) for antibody binding analysis from SPR machine (www.Biorad.com). Antibody titers were calculated using Prism 9.3.1 (GraphPad Software). All individual data is shown. The half-life (T1/2) of polyclonal binding antibodies for antibody decay was calculated using the nonlinear regression one-phase decay model in GraphPad Prism 9.3.1 (GraphPad Software).

For manuscripts utilizing custom algorithms or software that are central to the research but not yet described in published literature, software must be made available to editors and reviewers. We strongly encourage code deposition in a community repository (e.g. GitHub). See the Nature Portfolio guidelines for submitting code & software for further information.

Data

Policy information about availability of data

All manuscripts must include a data availability statement. This statement should provide the following information, where applicable:

- Accession codes, unique identifiers, or web links for publicly available datasets
- A description of any restrictions on data availability
- For clinical datasets or third party data, please ensure that the statement adheres to our policy

All data are shown in the manuscript figures and supplementary information. Source data are provided with this paper. There are restrictions to the availability of the GFPDL technology described in this study due to US patent application.

Research involving human participants, their data, or biological material

Policy information about studies with human participants or human data. See also policy information about sex, gender (identity/presentation), and sexual orientation and race, ethnicity and racism.

Reporting on sex and gender	All adults irrespective of sex were eligible for the study. Sex was not considered in the study design and study findings apply to both sexes. No sex-based analyses were performed in this study.
Reporting on race, ethnicity, or other socially relevant groupings	All adults irrespective of their race or ethnicity were eligible for the study. Race or ethnicity was not considered in the study design and study findings apply to all race or ethnicities. No race-based analyses were performed in this study.
Population characteristics	Participants in this study were adults in Uganda. The health status was self-reported. Sex, age, race, ethnicity, or previous health status was not considered in the study design and study findings apply to both sexes or any race or ethnicity or age. No sex-based or race-based analyses were performed in this study. Adult participants in these studies ranged in age from 18 to 54 years.
Recruitment	Recruitment was done at hospital for adult patients who came to the hospital with MARV infection or in the community for naive healthy adults who were eligible without any specific selection criteria or bias. Samples were collected following informed consent without any selection bias. Adult participants included confirmed MVD survivors, according to patient PCR and ELISA results, from the MARV outbreak of 2012 in Uganda, as well as healthy local community members that were not infected.
Ethics oversight	Human use protocols for collecting samples from MVD survivors in Uganda were reviewed and approved by the Helsinki committees of the Uganda Virus Research Institute in Entebbe, Uganda (reference number GC/127/13/01/15) and the Ugandan National Council for Science and Technology (registration number HS1332).<br>Samples were tested in different antibody assays with approval from the U.S. Food and Drug Administration's Research Involving Human Subjects Committee (FDA-RIHSC) under exemption protocol '15-064B'; and all assays performed fell within the permissible usages in the original consent.

Note that full information on the approval of the study protocol must also be provided in the manuscript.

Field-specific reporting

Please select the one below that is the best fit for your research. If you are not sure, read the appropriate sections before making your selection.

☒ Life sciences ☐ Behavioural & social sciences ☐ Ecological, evolutionary & environmental sciences

For a reference copy of the document with all sections, see nature.com/documents/nr-reporting-summary-flat.pdf

Life sciences study design

All studies must disclose on these points even when the disclosure is negative.

Sample size	All available samples were analyzed in this study.
Data exclusions	No data was excluded.
Replication	Neutralization, phage display, ELISA, FcR and SPR assays were performed twice by independent researchers in the lab. The replications were successful. The variation in duplicate experimental runs was <10% for neutralization, ELISA and FcR and <6% for SPR.
Randomization	All samples were analyzed in this study. The study was non-randomized. Initially, no patient information was provided, and all the immune analyses were conducted blindly by the researcher's performing the assays. The participants were assigned in each experimental group based on their MARV infection status and hospitalization (MVD survivors) or uninfected healthy controls.
Blinding	Experiments were performed by different investigators, who were blinded to sample identity.

Reporting for specific materials, systems and methods

We require information from authors about some types of materials, experimental systems and methods used in many studies. Here, indicate whether each material, system or method listed is relevant to your study. If you are not sure if a list item applies to your research, read the appropriate section before selecting a response.

Materials & experimental systems

n/a	Involved in the study
☐	☒ Antibodies
☐	☒ Eukaryotic cell lines
☒	☐ Palaeontology and archaeology
☐	☒ Animals and other organisms
☒	☐ Clinical data
☒	☐ Dual use research of concern
☒	☐ Plants

Methods

n/a	Involved in the study
☒	☐ ChIP-seq
☒	☐ Flow cytometry
☒	☐ MRI-based neuroimaging

Antibodies

Antibodies used	AlexaFluor 488 conjugated goat anti-mouse antibody was obtained from Invitrogen (Catalog # A28175) and used at 1:2000 dilution. (HRP)- conjugated goat anti-rabbit Fc fragment specific IgG from Jackson Immuno Research (Catalog number #111-035-046) was used at 1:5000 dilution.
Validation	Validation and testing for AlexaFluor 488 conjugated goat anti-mouse antibody was performed by the manufacturer - Invitrogen. Testing and validation for HRP- conjugated goat anti-rabbit Fc fragment specific IgG was performed by the manufacturer Jackson Immuno Research.

Eukaryotic cell lines

Policy information about cell lines and Sex and Gender in Research

Cell line source(s)	ATCC Vero E6 cell line was obtained from ATCC (Catalog number #ATCC-CRL-1586)
Authentication	The cell line was authenticated by karyotyping or other genomic techniques by ATCC
Mycoplasma contamination	Negative for Mycoplasma
Commonly misidentified lines (See ICLAC register)	No misidentified cell lines were used in the study.

Animals and other research organisms

Policy information about studies involving animals; ARRIVE guidelines recommended for reporting animal research, and Sex and Gender in Research

Laboratory animals	Female 4 to 6-weeks old New Zealand white rabbits.
Wild animals	Not applicable
Reporting on sex	Sex of rabbit species were not considered in study design. Only female NZW rabbits were included in the study, based on experience with previous studies in the lab on previous immunization studies have only used female NZW rabbits. Based on historical datasets and experience, we continued using the female NZW rabbits for immunization studies. It is expected that finding are applicable for animals of both sexes.
Field-collected samples	No field samples were collected. In labs, the rabbit study was carried out in strict accordance with the recommendations in the Guide for the Care and Use of Laboratory Animals of the National Institutes of Health and were performed under BSL2 conditions.
Ethics oversight	The rabbit experiments were approved by the U.S. FDA Institutional Animal Care and Use Committee (IACUC) under Protocol #2008-10. The animal care and use protocol meets National Institutes of Health guidelines.

Note that full information on the approval of the study protocol must also be provided in the manuscript.
