# Supplementary material for: Longitudinal proteome-wide antibody profiling in Marburg virus survivors identifies wing domain immunogen for vaccine design
Source: Nat Commun. 2024 Sep 17;15:8133. doi: 10.1038/s41467-024-51021-5 (PMC11405854; doi:10.1038/s41467-024-51021-5)
Supplement: Supplementary file 2 — Peer Review File [file 41467_2024_51021_MOESM2_ESM.pdf]

nature portfolio

Peer Review File

Longitudinal proteome-wide antibody profiling in Marburg virus survivors identifies conserved wing domain as target for vaccine design

Open Access This file is licensed under a Creative Commons Attribution 4.0 International License, which permits use, sharing, adaptation, distribution and reproduction in any medium or format, as long as you give appropriate credit to the original author(s) and the source, provide a link to the Creative Commons license, and indicate if changes were made. In the cases where the authors are anonymous, such as is the case for the reports of anonymous peer reviewers, author attribution should be to 'Anonymous Referee' followed by a clear attribution to the source work. The images or other third party material in this file are included in the article's Creative Commons license, unless indicated otherwise in a credit line to the material. If material is not included in the article's Creative Commons license and your intended use is not permitted by statutory regulation or exceeds the permitted use, you will need to obtain permission directly from the copyright holder. To view a copy of this license, visit http://creativecommons.org/licenses/by/4.0/.

Editorial Note: This manuscript has been previously reviewed at another journal that is not operating a transparent peer review scheme. This document only contains reviewer comments and rebuttal letters for versions considered at *Nature Communications.*

REVIEWERS' COMMENTS

Reviewer #2 (Remarks to the Author):

revisions and clarifications satisfy my comments

Reviewer #3 (Remarks to the Author):

The authors have provided a very comprehensive response to all my previous points. They have performed additional experiments and made numerous changes to the text that have addressed the majority of my initial concerns.

Response to Reviewer's comments

Reviewer #2 (Remarks to the Author):

revisions and clarifications satisfy my comments.

Reviewer #3 (Remarks to the Author):

The authors have provided a very comprehensive response to all my previous points. They have performed additional experiments and made numerous changes to the text that have addressed the majority of my initial concerns.

Response:

We thank both the reviewers for appreciating our revised manuscript.
